# What’s in a name? A discussion of what medical students and junior doctors call their senior colleagues, and why it matters

**DOI:** 10.15694/mep.2017.000163

**Published:** 2017-09-18

**Authors:** Janet Lefroy, Maggie Bartlett, Simon Gay, Ashley Hawarden, Ruth Kinston

**Affiliations:** 1 Keele University School of Medicine; 2 Nottingham School of Medicine

**Keywords:** Educational relationships

## Abstract

This article was migrated. The article was marked as recommended.

How should a medical student address their clinical tutor?

Sociolinguistic ideas such as politeness theory tell us that the choice of formal or informal terms of address is determined by the positions of those communicating on two axes; relative status and degree of intimacy. This positioning is influenced by the interaction of personal characteristics of the individuals involved, but there are cross-cultural variations to these rules which are also changing as the world changes. The purpose of the communication will also influence terms of address.

There is evidence that reducing social distance within teams improves team-working and that the perception of hierarchy prevents medical students asking for help. Such evidence forces us to take an honest look at how we train our junior colleagues to address us.

Students may discover that the etiquette of the medical school classroom differs from that of the clinical placement and find themselves uncertain about how to address their colleagues appropriately. We suggest that it may be helpful in such a quandary to ‘mind the gap’ rather than ignoring it or trying to close it by imposing a blanket rule on it.

We conclude by calling for sociological study with healthcare professionals and their students to discover whether formal or informal forms of address help or hinder aspects of learning and clinical teamwork.

## How should a medical student address their clinical tutor?

**Table:** 

“A person’s name is to that person, the sweetest, most important sound in any language” *How to win friends and influence people.* (Carnegie, 1936)

In using a person’s name the user has some influence over that person, if only to gain their attention and to claim a relationship. Names are intensely personal, and it is crucial to get this ‘most important sound’ ( Carnegie, 1936) right, but apart from pronunciation there are many social and cultural subtleties which have a profound effect on what is perceived to be right by those named.

**Table:** 

“Why should people I have never met, who read me in bed and in the bathtub, think of me as ‘Sam’?” Samuel (‘Sam’) Schoenbaum insisting that his first name be represented by the initial ‘S’ on the title pages of his books (Times obituary 25 ^th^ April 1996.)

Sociolinguistic study tells us that the choice of formal or informal terms of address is determined by the position of those communicating on two axes - relative status and degree of intimacy. Age, gender, occupation, family ties and emotional solidarity are relational characteristics which will influence this positioning, but there are cross-cultural variations in the social distance and power distance engendered by these characteristics. ( Martiny, 1996; D. a Morand, 2003) The purpose of the communication will also influence terms of address as more threatening communications will require more politeness in order to be accepted. (D. A. Morand, 2000) Politeness theory posits that subordinates use greater amounts of polite language, which includes honorifics such as ‘sir’ or ‘Dr’, than do superiors. ( Brown & Levinson, 1987; Nevala, 2004) The explanation is that subordinates are careful not to offend their superiors (for example by seeming to lack deference or claim over-familiarity by using a first name) especially when dependent on them for something and therefore needing to keep goodwill flowing. Conversely however, there is evidence that reducing social distance within teams improves team-working, and that the perception of hierarchy prevents medical students asking for help. ( Smith, Tallentire, Cameron, & Wood, 2013)

Each of the authors has a different code of practice when it comes to what we allow or prefer students to call us. JL suggests they call her ‘Janet’ because that is what her non-medical colleagues do with student groups from year 1, believing this to emphasise adult learner and collegiate status. MB allows students to call her ‘Maggie’ when apprenticed to her in General Practice where that is what all the staff call her, but in the school it is ‘Dr Bartlett’ as she has a disciplinary role as co-lead of final year. SG also prefers to be addressed as ‘Dr Gay’ until students qualify whereupon part of their transition to being a doctor is marked by having permission to call him ‘Simon’. Meanwhile, on hospital placements students may encounter consultants who will not expect to be on first name terms until a colleague reaches senior registrar level or above. RK by contrast is one hospital consultant who doesn’t feel strongly about what students or colleagues call her, and is intrigued as to why some decide to call her Ruth and others call her Dr Kinston.

The junior colleague perspective is provided by AH who, despite having co-authored research with JL and SG has declined the invitation to call them by their first names and still addresses the other authors by title two years after graduation. This is a conscious decision in recognition of the status of those he views as authoritative figures. In his undergraduate years there was more uncertainty and a fear of causing offence and even now addressing a senior researcher by their first name would feel somewhat uncomfortable. AH perceives these views to be driven at least partially by experiences as a student and junior doctor working within a hospital environment when he became aware that an unwritten social hierarchy exits. His response was to call all senior clinicians (generally consultants and registrars from other clinical teams) by their title. This ensures that one does not cause any offence in a culture where impressions and respectfulness are highly valued.

RK followed the same rules when she was a student. As a pre-clinical student in the late 80’s/early 90’s there was never any question regarding what one should call tutors. Titles were formal and strictly adhered to. When working a student locum however, she noted that as a team member, she was allowed to call the medical registrar by his first name. She discovered that this helped her confidence and reduced nervousness about approaching him for advice. She noticed that he also used his first name with the nurses and this again broke down barriers. His approachability and the fact that he was also an excellent clinician inspired her. When she qualified she considered how she wished to be perceived by patients and indeed staff. Feeling no more capable on day one after qualifying, she felt uncomfortable calling herself ‘doctor’. She adopted a halfway house and introduced herself as ‘Ruth Kinston I’m your junior doctor’. This approach has continued to suit her as the years have passed. She now says ‘Hello my name is Ruth Kinston, I’m the consultant doctor’ and is happy if they then call her Ruth. She is equally happy to be called Dr Kinston by patients, students and colleagues if they prefer and some seem to. She considers that this is no block to being taken seriously when it matters, or to leading a team.

The unwritten rules (hidden curriculum) surrounding hierarchy influences students of all health professions from their induction into the NHS. It is possibly stronger in hospital than in general practice although many GPs prefer patients to call them ‘Doctor’ to emphasise the professional relationship they have. In turn some, but not all, who like to be called ‘Doctor’ will also use titles when addressing patients.

This is all taking place within the milieu of broader, accelerated, societal and cultural trends. Whereas 100 years ago in the UK everyone had a title (reflecting the strength of social hierarchies), modern society is less formal, less hierarchical and people are more likely to be on first name terms. In some parts of the world this is even more the case (such as in Australia). In others it is less (such as in Germany, where professionals have two titles, ‘Frau’ or ‘Herr’ ‘Doktor’, and ‘Doktor’ by itself is considered disrespectful). In many African countries there is yet another norm - it would be disrespectful to use the name of a senior person without a title but the name used could be their given name (or that of the person’s oldest child). So when living and working in Tanzania JL was Mama Benjamin or Doctor Janet and could be Mama Janet but never Janet. The mixture of familiarity and respectfulness this created was an acceptable compromise in that culture.

The problem of what our students should call us in a multi-ethnic NHS is further compounded by the difference we have mentioned between the etiquette of the medical school classroom and that of the clinical placement. Some students inadvertently transgress codes by assuming that they can call their consultant by their first name, having learned the habit in medical school small group tutorials.

Is it any wonder that students are uncertain how to address their colleagues appropriately in any given context?

We therefore give guidance to our first year students, suggesting that if they are in doubt as to how to address tutors (or patients), they are best to choose the formal address. If the tutor then says “call me Ravi” that is what they may do. If it seems appropriate (e.g. introductions are being done and the placement is several days long) they might ask politely how they should address the tutor and the other members of the team.

This advice does not however help the student who feels uncomfortable calling a doctor “Ravi” and wishes to remain at a respectful distance, nor does it help the student who feels constrained by the hierarchy implied by differing titles. When faced with such a difference it can be very helpful to students to ‘mind the gap’ rather than ignoring it or trying to close it by imposing a blanket rule on it. Minding the gap involves:

• Acknowledging the difference. As tutors we may assume such matters are minor and easily reconciled but from the learner’s viewpoint it may look insurmountable. Kneebone’s comparison with a ‘ha-ha’ ditch and wall is apt. ( Kneebone, 2009)
• Using the difference as a focus for constructive learning rather than favouring the hidden curriculum. Discussing differences they notice, working out why they exist and deciding what meaning and action they will take as a result can train the students to do their own reflecting in future. ( Yardley, Irvine, & Lefroy, 2013)

Having discussed and reviewed the use of names and titles in the complex ‘swampy lowlands’( Schon, 1991) of medical school classroom and placement etiquette from the perspective of the clinician tutors, the authors have been struck by the lack of detailed information from the student perspective, which is arguably the most important perspective of all. We don’t really know how big a problem this inconsistency and complexity is for students, how much of a barrier it poses to their learning, how much of their emotional energy it consumes or even how students actually deal with it on a practical level on a daily basis. It may be worthy of wider sociological study with healthcare professionals and their students.

**Table:** 

“What’s in a name? That which we call a rose By any other name would smell as sweet.” Romeo and Juliet, 1595. Act 2, Scene 2. William Shakespeare ( The Oxford Shakespeare, 1914)

## Take Home Messages

• Students may discover that the etiquette of the medical school classroom differs from that of the clinical placement and find themselves uncertain about how to address their colleagues appropriately
• Such norms are culturally determined and are changing
• There is evidence that reducing social distance within teams improves team-working and that the perception of hierarchy prevents medical students asking for help
• Students are advised to err on the formal side when in doubt, but both they and their seniors would do well to ‘mind the gap’

## Notes On Contributors

JL is a senior lecturer in medical education and lead for consultation skills at Keele University School of Medicine. She is also a general practitioner. Her interests include workplace assessment and feedback, and transitions from medical school to clinical practice.

MB is a clinical senior lecturer in medical education, Deputy Director of Education in General Practice and a final year co-lead at Keele University School of Medicine. Her interests include quality in community based teaching and cognitive load in simulation.

SG is Director of Education Governance and Clinical Associate Professor in Medical Education at the University of Nottingham Medical School and Undergraduate Curriculum Advisor at Keele University School of Medicine. His current research interests include clinical reasoning, skills development, and professionalism

AH is a clinical teaching fellow at Keele University School of Medicine. His main interests include assessment and feedback.

RK is an emergency medicine consultant at the Royal Stoke University Hospital and a final year co-lead at Keele University school of medicine. Her interests include simulation and preparedness for clinical practice.

JL, SG and MB had the initial discussion which spawned this commentary. JL wrote the first draft which was developed and edited by SG and MB. AH and RK were recruited to provide their individual perspectives and all authors revised the final drafts.
