# Supplementary material for: What’s in a name? A discussion of what medical students and junior doctors call their senior colleagues, and why it matters
Source: MedEdPublish (2016). 2017 Sep 18;6:163. [Version 1] doi: 10.15694/mep.2017.000163 (PMC10885294; doi:10.15694/mep.2017.000163)
Supplement: Peer review history

# Reviewer report: Reviewer response for version 1

**DOI:** 10.21956/mep.19348.r28282

**Published:** 2017-09-24

**Authors:** Trevor Gibbs

**Affiliations:** 1 AMEE

This review has been migrated. The reviewer awarded 4 stars out of 5 An interesting paper that addresses a potentially difficult area- I felt that it was very thought provoking and in this era of changing and challenging cultures, a subject we need to address. I would support the authors call for an in-depth sociological exploration of the subject

Reviewer Expertise:

NA

No decision status is available

# Reviewer report: Reviewer response for version 1

**DOI:** 10.21956/mep.19348.r28280

**Published:** 2017-09-18

**Authors:** James Fraser

**Affiliations:** 1 Griffith University

This review has been migrated. The reviewer awarded 4 stars out of 5 This paper addresses an interesting topic and by considering potential impacts on student learning add a new layer to the hidden curriculum in medical programs. Even in Australia where we are ‘more likely to be on first name basis’ there is similar variation in terms of address to those described in the paper. The paper raises interesting questions for future work regarding the impact of these variations on learning, power differential and the culture in clinical setting and how that in turn impacts on student learning. The paper is a good trigger for personal reflection on our teaching practice.

Reviewer Expertise:

NA

No decision status is available

# Reviewer report: Reviewer response for version 1

**DOI:** 10.21956/mep.19348.r28279

**Published:** 2017-09-18

**Authors:** John Dent

**Affiliations:** 1 AMEE

This review has been migrated. The reviewer awarded 4 stars out of 5 What an unexpected and yet very relevant topic! This paper raises the interesting question of what name we prefer to be called by our students and suggests that student uncertainly on the answer to this question may be an under-recognised source of difficulties in relationship or education. The discussion opens up questions of politeness theory, perceptions of status, intimacy and etiquette as well as personal characteristics which are each engaging and useful.I think this paper raises a new topic for the consideration of all tutors and clinicians in regard to how they can best relate to students. It will be interesting to read further comments from other readers.

Reviewer Expertise:

NA

No decision status is available

# Reviewer report: Reviewer response for version 1

**DOI:** 10.21956/mep.19348.r28281

**Published:** 2017-09-18

**Authors:** P Ravi Shankar

**Affiliations:** 1 American International Medical University

This review has been migrated. The reviewer awarded 4 stars out of 5 This is an interesting and well-written paper on an important topic which is not often discussed. I have been in a number of countries during the course of my education and teaching career. In the 1980s and 1990s in India during my medical education relationships were formal and we addressed male teachers as ‘Sir’ and female teachers as ‘Madam’. We rarely used any first names and this was considered rude and discouraged by our teachers. In Nepal again relationships were hierarchical and I was always addressed as Sir though I did make lot of efforts to bridge the distance. I have been involved in facilitating small group sessions and would have been preferred to be addressed as Dr Ravi.In Aruba in the Caribbean we had an interesting situation. Most of our students were second-generation Americans or Canadians whose parents were from Asia. They mostly addressed me as ‘Dr Ravi’ but did occasionally use the term ‘Sir’. In Saint Lucia in the Caribbean the students are mainly from South Asia and West Africa and again I am addressed as ‘Sir’. As the authors point out the term of address can influence the power balance in the relationship and how the learners regard their teachers/facilitators.

Reviewer Expertise:

NA

No decision status is available
